# Extracorporeal liver support: trending epidemiology and mortality - a nationwide database analysis 2007–2015

**DOI:** 10.1186/s12876-019-1077-y

**Published:** 2019-09-03

**Authors:** Thomas Wiesmann, Dominic Hoenl, Hinnerk Wulf, Marc Irqsusi

**Affiliations:** 1 Department of Anesthesiology and Intensive Care Medicine, Faculty of Medicine, University Hospital Marburg, Philipps University, Baldingerstrasse, 35033 Marburg, Germany; 2 Department of Cardiothoracic Surgery, University Hospital Marburg, Philipps University, Baldingerstrasse, 35033 Marburg, Germany

**Keywords:** Extracorporal liver support, Extracorporal liver replacement, Liver assist device, Acute liver failure, Liver transplantation

## Abstract

**Background:**

Extracorporeal liver support therapies (ELS) are technical options (for bridge-to-recovery as well as bridge-to-transplant) in patients with acute liver dysfunction (e.g. acute liver failure (ALF), acute-on chronic liver failure (AoCLF) or decompensated chronic liver disease (decomp. CLD)) to reduce effects of failing hepatic detoxification functions. The present study investigates the real-life utilization of ELS (annual incidences), mortality rates as well as data regarding specific populations of liver transplantation in Germany.

**Methods:**

Data on patient cases receiving extracorporeal liver support therapy were identified in a nationwide data set from the Federal statistical Office of Germany from 1 January 2007 through 31 December 2015 and analyzed regarding in-hospital mortality, age- and sex-specific distribution and use of ELS in the context of liver transplantation. Mortality rates in patients with primary acute liver dysfunction and secondary acute liver dysfunction (in the context of cardiothoracic surgery) were evaluated.

**Results:**

Annual incidences of ELS use remained stable between 0.39/100.000 in 2007 and 0.47/100.000 ELS in 2015. In-hospital mortality rate was 51.49% in the 2886 evaluated patient cases. Mortality was higher in men (56.04%) than in women (43.70) in the observed time period between 2007 and 2015. ELS utilization and case-related liver transplantation rates were low (12.47%). Since 2012, the annual numbers for ELS therapy in cardiosurgical patients exceeded the frequency of ELS utilization in cases of primary liver dysfunction (mortality rates: 68.39% versus 40.63%).

**Conclusions:**

ELS utilization remained stable between 2007 and 2015. Mortality rates are high in this patient population of acute liver dysfunction, especially in combination with case-related cardiothoracic surgery. ELS is rarely used in the setting of liver transplantation. In 2015, more than 50% of all ELS cases in Germany were performed in the context of cardiothoracic surgery.

## Background

Acute liver dysfunction might occur in patients with preexisting (acute- on-chronic, AoCLF; decompensated chronic liver disease) or non-preexisting (acute liver failure, ALF) liver disease [1, 2]. Similar to other extracorporeal therapies for dedicated organ failures (e.g. dialysis for acute kidney failure, ECMO for severe lung and/or heart failure), extracorporeal liver support (ELS) is an invasive option to mitigate the effects of failing liver functions [3–6]. Ideally, this approach should result in providing of the whole range of liver functions including detoxification, synthesis, excretion, metabolic aspects and other regulatory functions [7]. There are two different types of artificial/ extracorporeal liver support therapy that might be subclassified as artificial or bioartificial (bioreactor) types which are mainly focussing on detoxification and metabolic stabilization. Bioartificial liver support systems use living cells (porcine or human-derived) loaded in an extracorporeal bioreactor. Currently, there is no bioartificial system clinically available outside clinical trials [7, 8]. Regarding the artificial liver support options, blood purification is the main principle used in various approaches (Molecular adsorbent recirculating system, (MARS®, Gambro, Lund, Sweden); FPSA, fractionated plasma separation, adsorption, and dialysis technology of the Prometheus® System (Fresenius Medical Care, Bad Homburg, Germany; single pass albumin dialysis (SPAD) as well as the principle of plasmapheresis/ plasma exchange) [7, 8]. Several prospective trials investigated the effects of the first two mentioned commercially available extracorporeal devices. Only few studies showed a trend towards decreased mortality in patients receiving ELS and improvement of hepatic encephalopathy compared to conventional therapy [9, 10]. However, clear evidence of relevant clinical benefits in specific subpopulations of liver dysfunction is still missing [9]. Furthermore, ELS therapy is performed at some centers in patients with acute liver dysfunction (in acute liver failure and in graft dysfunction [11, 12]) as a bridging option (bridge-to-transplant or bridge-to-recovery). Extracorporeal liver support therapies are not only performed in patients with primary liver dysfunction but also increasingly performed by some centers in cases of secondary liver failure (in the absence of prospective studies), e. g. hypoxic hepatitis (acute ischemic hepatocellular injury) in patients with previous cardiac surgery or with veno-arterial extracorporeal life support (ECLS/ VA-ECMO) devices [2, 6, 13–15]. For these particular indications, no prospective study data has been published in international literature.

Aim of this study was to investigate the epidemiologic development of ELS utilization and evaluate real-life mortality in a high-income country from 2007 to 2015. In detail, age distribution, sex specific mortality rates, causes of underlying liver dysfunction (primary or secondary) as well as mortality rates of patients with ELS and primary or secondary liver failure were points this study intended to address. Furthermore, we investigated clinical utilization of ELS as a bridge-to-transplant or graft-failure option through examination of the performed evaluation steps for liver transplantation as well as successful transplantation numbers in ELS patients.

## Methods

### Data source

We performed a database analysis using data provided by the Federal Statistical office in Germany (Statistisches Bundesamt, Wiesbaden, Germany). We received permission by this institution to publish this data within our manuscript. Thus, no further institutional review board approval was required. The German health care system records and documents all patient cases of hospital treatments according to the German accounting method (German DRG) as main diagnosis according to ICD (International Statistical Classification of Diseases and Related Health Problems) as well as further diagnoses and performed case-related operations and procedures (OPS).

### Cohort definition

As ELS treatments are only performed in a hospital setting, we were able to provide information on the total annual German case numbers. Procedures performed during the hospital stay were documented according to the current OPS coding (operation & procedure key codes). Case related data of the respective diagnosis and OPS were transferred from hospitals or insurance companies to the federal statistical office according to national laws.

### Primary & secondary endpoints

A database query was performed for the given years regarding patient cases with the respective OPS code for “extracorporeal liver assist device” (“liver dialysis”, OPS 8–858, Table 1) and the case-related mortality of these patients overall as well as grouped by sex and stratified age spans. To our knowledge, in the studied time span from 2007 to 2015, only the “molecular adsorbent recirculating system” (MARS, Gambro, Lund, Sweden) as well as “fractionated plasma separation and adsorption” system (FPSA, Prometheus, Fresenius Medical Care, Bad Homburg, Germany) were commercially available in Germany. The OPS coding for plasmapheresis (8–820) was not investigated as this coding is not specific enough to identify patients with hepatic failure who underwent therapeutic plasma exchange. The statistics office registered a population of 82,175.684 (as of 31.12.2015) people with minor changes during the last 10 years in Germany. For annual incidence calculations (primary endpoints), the statistical population of each corresponding year was used. The patient’s status at discharge (e.g. in-hospital death, transfer to another hospital, discharge to a specific care facility, discharge to home) is documented for each case with the German DRG system. Thus, we were able to calculate in-hospital mortality rates for ELS cases and some specific subpopulations.

**Table 1:** Case definitions

Disease / Procedure	Specification	ICD / OPS coding
Extracorporal liver support therapy	“Liver dialysis”	OPS 8–858
Liver transplantation	Liver transplantation (all variants)	OPS 5–504
Evaluation for liver transplantation		
“Evaluation & no waiting list”	Complete evaluation, without inclusion of a patient on an organ transplantation waiting list	OPS 1–920.04
	Partial evaluation without inclusion of a patient on an organ transplantation waiting list	OPS 1–920.14
“Evaluation & waiting list”	Complete evaluation, with inclusion of a patient on an organ transplantation waiting list	OPS 1–920.24
“Reevaluation”	Re-evaluation, with inclusion or retention of a patient on an organ transplantation waiting list	OPS 1–920.34
	Re-evaluation, with removal of a patient from an organ transplantation waiting list	OPS 1–920.44

*ICD* International Classification of Disease, *OPS* Operations & Procedures Coding. For details see text

Additional analysis (secondary endpoints) was performed to estimate the real-life use of ELS in combination with bridge-to-transplant therapy or for graft dysfunction (temporal sequence cannot be evaluated within the data set as only case-specific coding of disease and procedures are given by the Federal Statistical office). Thus, OPS codes of ELS and performed liver transplantation (OPS 5–504) or evaluation for liver transplantation (OPS 5–504 within the same case of ELS) were analyzed (Table 1).

Another subgroup analysis was performed to evaluate subpopulations of patients with primary (and therefore potentially eligible for liver transplant) and secondary acute liver failure. Regarding the secondary acute liver dysfunction, we decided to explore the utilization of ELS technology in patients with acute liver failure in the context of major cardiac surgical procedures as several studies suggested an increase of ELS usage within this population during the last decade [6, 13, 14].

Using typical ICD coding of acute primary liver failure with typical indications for liver transplantation according to Eurotransplant criteria [16], a “primary liver failure group” was established and mortality rates were calculated within this patient subpopulation during the given years (2007–2015). These mortality rates were set against the mortality rates of another patient subpopulation with ELS and OPS-codes related to cardiac surgery within the same case (e.g. coronary, valvular or aortic surgical procedures) to establish an exemplary group of patient population with secondary liver failure. All OPS and ICD-10-GM codes used in this study are listed in the Tables 1 and 2.

**Table 2:** Subgroup analysis primary vs. secondary liver failure & ELS – Parameters & definitions

“Primary liver failure group”	
Alcoholic liver disease	ICD K70
Liver failure, not elsewhere classified (includes fulminant liver failure)	ICD K72
Fibrosis and cirrhosis of the liver	ICD K74
Other diseases of the liver	ICD K76
Other diseases of the biliary tract	ICD K83
Malignant neoplasm of liver and intrahepatic bile ducts	ICD C22
Congenital malformations of the gallbladder, bile ducts and liver	ICD Q44
Disorders of mineral metabolism (includes M. Wilson, Hemochromatosis)	ICD E83
Other metabolic disorders	ICD E88
Other venous embolism and thrombosis (including Budd-Chiari-Syndrome)	ICD I82
“Secondary liver failure group” – Cardiac surgery patient population	
Operative external circulation (when using the heart-lung machine)	OPS 8–851
Operations on valves and septa of the heart and cardiac vessel	OPS 5–35
Surgery on the coronary vessels	OPS 5–36
Rhythm surgery and other operations on the heart and pericardium	OPS 5–37
Resection and replacement (interposition) at the aorta	OPS 5–384

Primary liver failure group coding was chosen according to typical criteria by EuroTransplant for performed non-high-urgency liver transplantation (See reference [12], Jochman et al.). Secondary liver failure group (“Cardiac population”) was chosen according to predefined cardiac surgical procedures (coronary, valvular and aortic surgery performed in combination with heart-lung machine)

### Patient involvement

Patients were not involved in the research questions, outcome measures or other study-related issues.

### Reporting guidelines

We followed the STROBE recommendations for adequate reporting of observational routine data.

### Statistical analysis

Data were analyzed descriptively. Absolute numbers and mortality rates were stratified for different age groups and by sex and year. Annual incidences were calculated as described above. Excel for Mac (Release 15.32, Microsoft Corp., Seattle, WA, USA) and SPSS (release 22, IBM SPSS, Armond, NY, USA) were used for analysis.

## Results

### Primary endpoints: annual incidences & in- hospital mortality rates

A total of 2886 patient cases with extracorporeal liver support (ELS) was documented in the German nationwide database in the observed time-frame from 2007 until 2015.

**Fig. 1:**
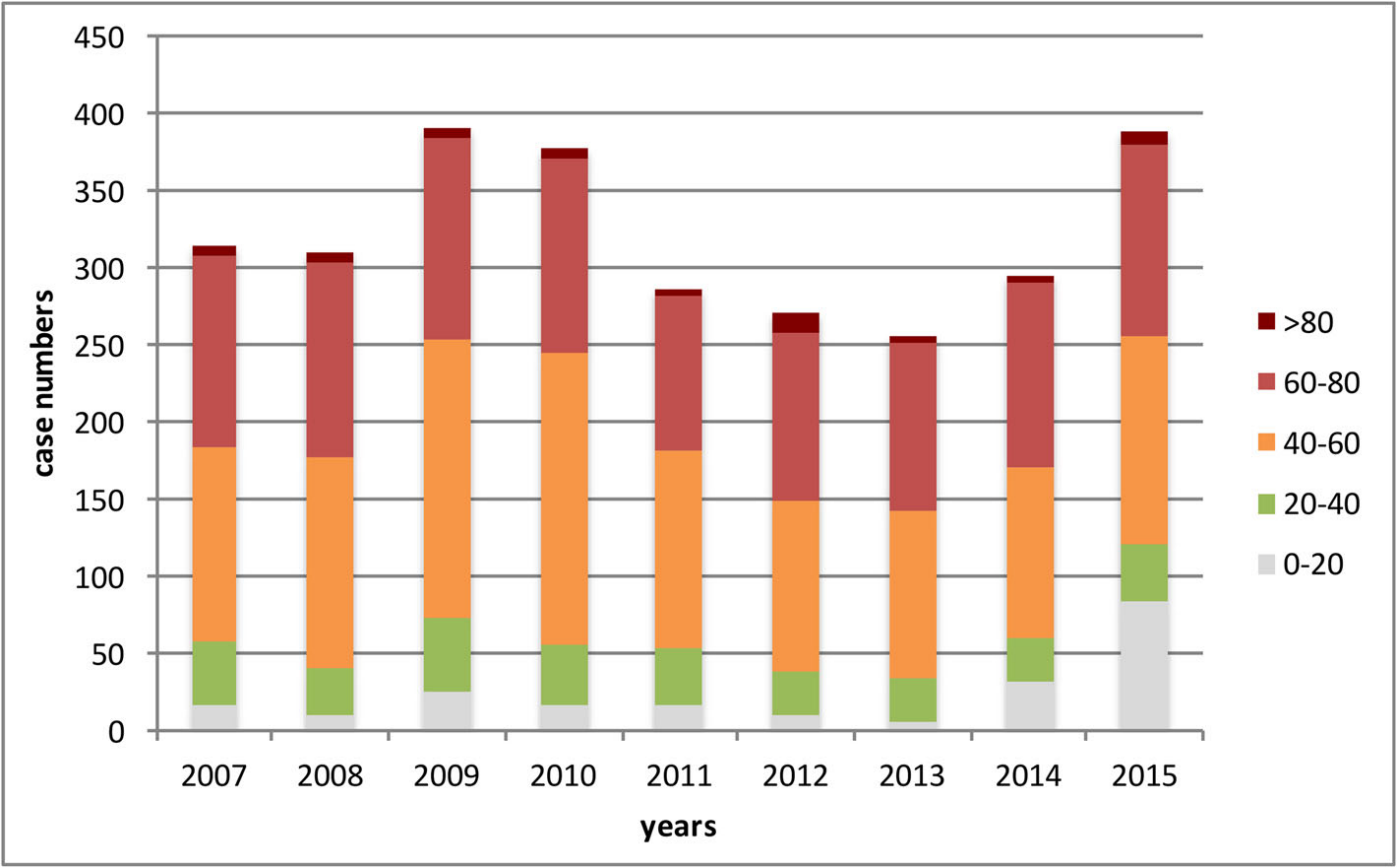
Extracorporeal Liver Support Therapy – Age groups

The annual numbers of ELS ranged from 205 (in 2013) to 390 cases (in 2009). This represents an incidence of 0.39/100.000 inhabitants in 2007 and 0.47/100.000 inhabitants in 2015. Overall mortality was 51.49% for the 2886 investigated patients (Table 3). The majority of all ELS therapies were performed in the age groups 40–60 and 60–80 years (79.42%) (Table 3 and Fig. 1).

**Table 3:** Epidemiology of Extracorporeal liver support (ELS) utilization in Germany

		2007	2008	2009	2010	2011	2012	2013	2014	2015	Overall 2007–2015
All ELS patients											
	n	314	311	390	377	287	270	255	294	388	2886
	Mortality (%)	57.32	55.63	46.41	46.42	52.61	55.18	61.18	50.00	44.84	51.49
Annual incidence	(n/100.000)	0.39	0.39	0.48	0.47	0.36	0.34	0.32	0.36	0.47	0.43
Sex											
Male	n	211	191	247	238	190	176	171	179	219	1822
	Mortality (%)	60.19	57.59	51.01	50.84	55.26	54.55	62.57	55.87	58.90	56.04
Female	n	103	120	143	139	97	94	84	115	169	1064
	Mortality (%)	51.46	52.50	38.46	38.85	47.42	56.38	58.33	40.87	26.63	43.70
Age groups											
0–20 years	n	17	10	25	17	16	10	5	32	84	216
20–40 years	n	41	30	49	38	37	28	29	29	38	319
40–60 years	n	126	137	180	189	130	111	109	109	134	1225
60–80 years	n	124	126	129	126	99	109	109	121	124	1067
>80 years	n	6	8	7	7	5	12	3	3	8	59

Data is presented as absolute numbers and percentage. ELS, extracorporal liver support. N, number. Incidences were calculated by using the year-specific population numbers for Germany as provided by the Federal Statistical Office. Due to data protection restrictions, further detailed analysis was not permitted by the data source provider, German Federal Statistical Office (e.g. age-specific mortality rates). For details, see text

**Fig. 2:**
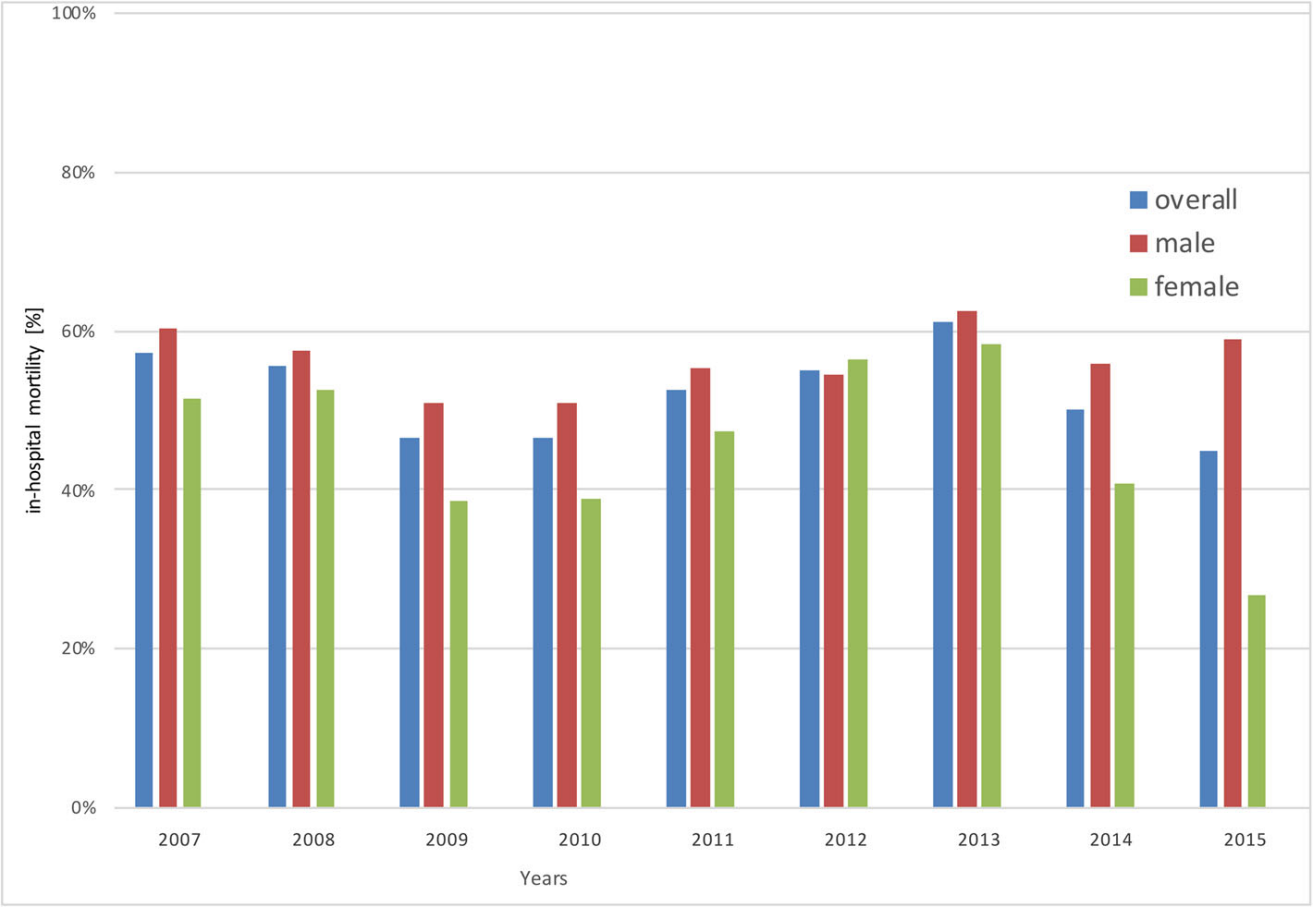
Extracorporeal Liver Support Therapy – Sex specific mortality

Absolute numbers for male patients receiving ELS were higher than in females from 2007 through 2015 (1822 male vs. 1064 female). Additionally, sex-specific mortality rates were higher for males (56.04%) compared to female patients (43.70%) receiving extracorporeal liver support therapy (Fig. 2).

### Secondary endpoints: ELS & liver transplantation

**Fig. 3:**
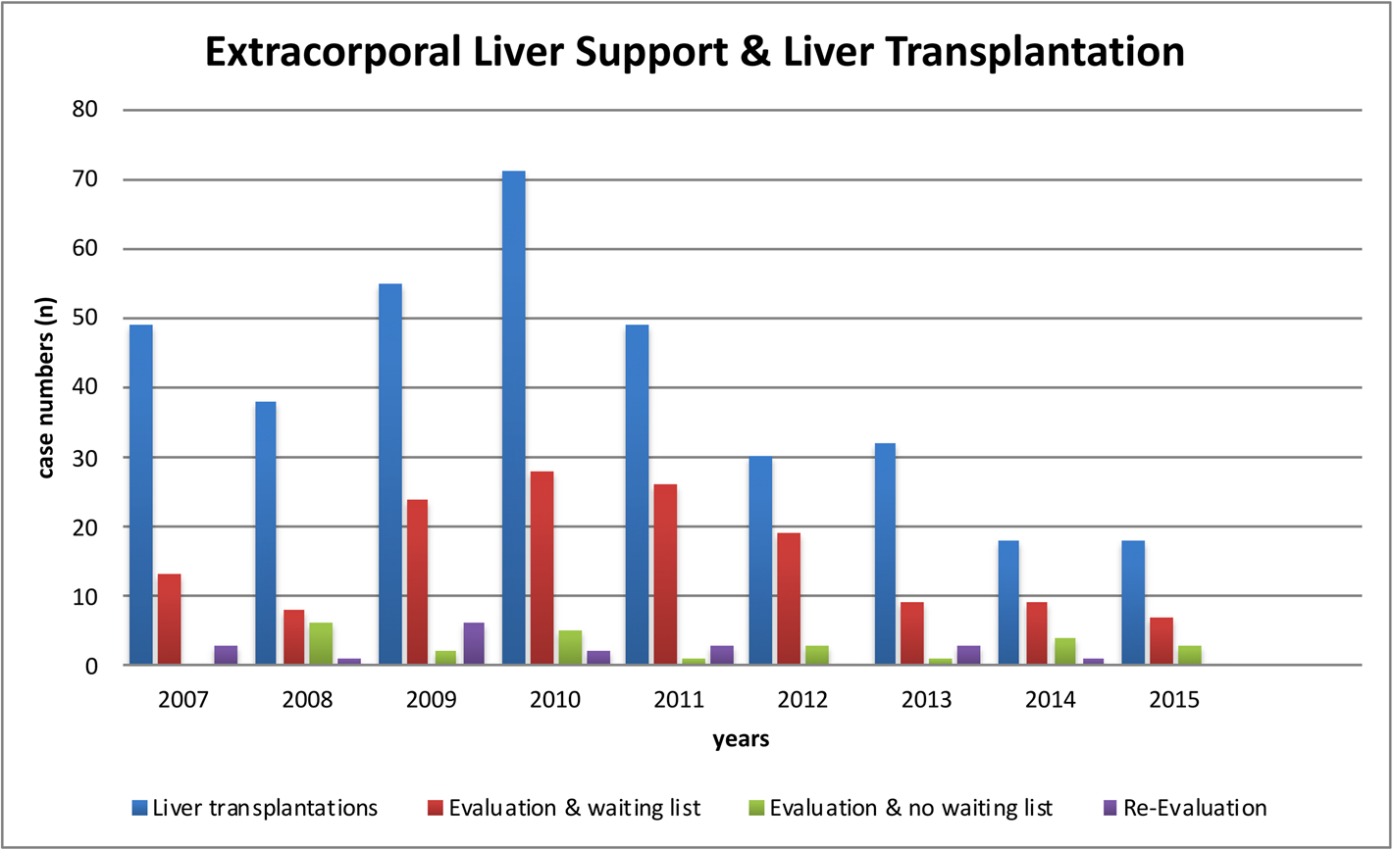
Utilization of Extracorporeal Liver Support Therapy & liver transplantation

Overall, 360 of the 2886 patients (12.4%) received ELS as a peri-transplantation therapy option (before (“bridge-to-transplant”) or after liver transplantation (due to graft dysfunction) within the same case) between 2007 and 2015. Forty-nine liver transplantations were performed in 2007 and 18 in 2015 within the observed ELS patient cases (Table 4). Mortality rate of patients who received ELS as a peri-transplantation therapy between 2007 and 2015 was 37.5% (135 of 360 patients. Evaluation with listing for transplantation was performed in 143 patients (4.95%) whereas evaluation without waiting list placement was made in 25 patients (0.87%) between 2007 and 2015 (Fig. 3). For comparison, a total of 8766 liver transplantations were performed in Germany between 2007 and 2015 according to the data source provider.

**Table 4:** Liver transplantation and (case-concomitant) transplant evaluation & performed ELS therapy

	2007	2008	2009	2010	2011	2012	2013	2014	2015	2007–2015
Liver transplantations, *n* (%)	49 (15.6)	38 (12.2)	55 (14.1)	71 (18.8)	49 (17.1)	30 (11.1)	32 (12.5)	18 (6.1)	18 (4.6)	360 (12.4)
In-hospital Mortality	19 (38.8)	22 (57.9)	17 (30.9)	23 (32.4)	15 (30.6)	8 (26.7)	20 (62.5)	4 (22.2)	7 (38.9)	135 (37.5)
Evaluation & waiting list, *n* (%)	13 (4.14)	8 (2.57)	24 (6.15)	28 (7.43)	26 (9.06)	19 (7.04)	9 (3.53)	9 (3.06)	7 (1.80)	143 (4.95)
Evaluation & no waiting list, *n* (%)	0	6 (1.92)	2 (0.51)	5 (1.33)	1 (0.35)	3 (1.11)	1 (0.39)	4 (1.36)	3 (0.77)	25 (0.87)
Re-Evaluation, *n *(%)	3 (0.96)	1 (0.32)	6 (1.54)	2 (0.53)	3 (1.05)	0	3 (1.18)	1 (0.34)	0	19 (0.66)

Data given as absolute numbers and percentage values of all ELS cases of the respective year. Documentation of transplant evaluations might underlie a relevant underreporting bias as numbers of performed liver transplantations are higher than documented evaluations for transplantation. The data source does not allow for differentiation if ELS therapy was used before or after liver transplantation due to national data protection laws. For details see text & Table 1

### ELS in primary liver dysfunction versus secondary liver dysfunction (in the context of cardiac surgery)

**Fig. 4:**
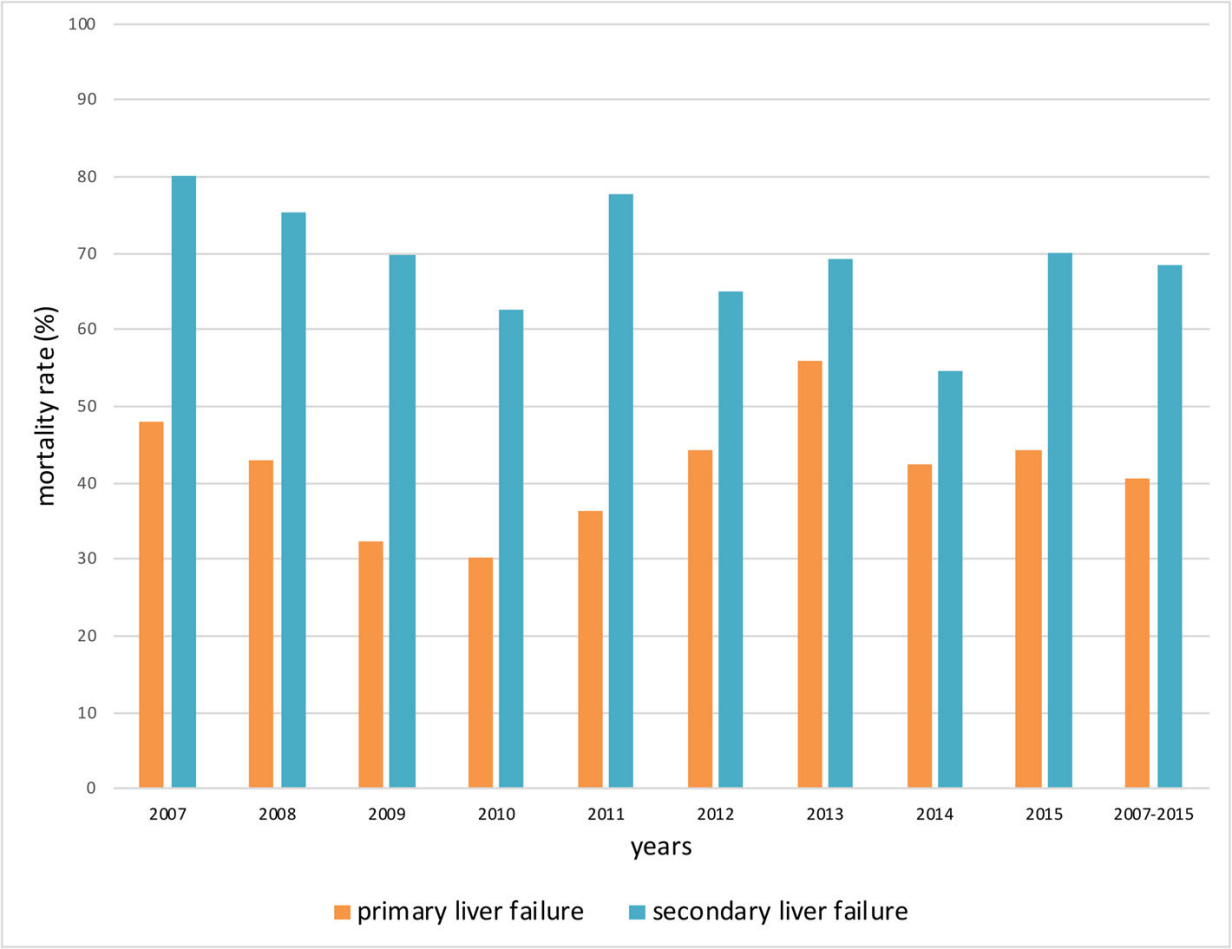
Comparison of mortality rates in primary versus secondary acute liver dysfunction

Since 2012, the annual ELS therapy numbers in the context of cardiac surgery cases exceeded the numbers of acute liver dysfunction as defined in our study population (2015: 196 cases (50.52% of all ELS cases) with cardiac surgery OPS coding versus 131 cases (33.76% of all ELS cases) with a typical ICD diagnosis for primary liver failure (Table 5 and Fig. 4). Mortality rates in ELS cases in the cardiac surgery group were higher compared to the primary liver dysfunction group (68.39% vs. 40.63% between 2007 and 2015).

**Table 5:** Extracorporeal liver support therapy in primary vs. secondary (cardiac surgery) liver failure

	2007	2008	2009	2010	2011	2012	2013	2014	2015	2007–2015
Overall ELS therapies, n	314	311	390	377	287	270	255	294	388	2886
Mortality (%)	57.32	55.63	46.41	46.42	52.61	55.18	61.18	50.00	44.84	51,49
Primary ALS, n (% of all ELS)	152 (48.41)	156 (50.16)	182 (46.67)	189 (50.13)	132 (45.99)	97 (35.93)	102(40.00)	92 (31.29)	131 (33.76)	1233 (42.72)
Mortality (%)	48,03	42,95	32,42	30,16	36,36	44,33	55,88	42,39	44,27	40,63
Secondary ALS, n (% of all ELS)	115 (36,62)	129 (41,48)	126 (32,31)	139 (36,87)	121 (42,16)	157 (58,15)	140 (54,90)	190 (64,63)	196 (50,52)	1313 (45,50)
Mortality (%)	80,00	75,19	69,84	62,59	77,69	64,97	69,29	54,74	69,90	68,39

Data given as absolute numbers and percentage values of all ELS cases of the respective year. ELS, extracorporeal liver support; n, number. Further differentiation (age, sex) was not permitted in this subgroup analysis due to national data protection laws. For definitions of primary and secondary liver failure see text & Table 2

## Discussion

This is the first epidemiological study investigating real-life utilization of extracorporeal liver support therapy in a high-income country (Germany) from 2007 through 2015. The main findings are:

Use of extracorporeal liver support therapy in Germany remained stable during the observed period. A high mortality in the population was shown, potentially due to the underlying diseases. Mortality was higher in male than in female patients with ELS. Since 2012, the annual numbers of ELS therapies in patients with case-concomitant cardiothoracic surgery exceeded the numbers of ELS utilization in cases of typical acute primary liver dysfunction.

Extracorporeal liver support therapy before and after liver transplantation was only a marginal aspect in clinical practice according to our real-life data. Mortality in patients treated with ELS due to primary causes of acute liver dysfunction was lower than in patients with case-concomitant cardiothoracic surgery.

Our real-life data study shows a higher in-hospital mortality in patients who received extracorporeal liver support therapy compared with the mortality rates of patients who received ELS treatment in randomized controlled clinical trials, e.g. a 28-day mortality of 58% in the RELIEF trial (ACLF population) [9], 30-day mortality of 9.5% in the FULMAR study (ALF patients on high-priority national wait list for liver transplantation) [17] and a 28-day mortality of 34% in the ACLF study by Kribben et al. [18]. This might be explainable by the fact, that the RCTs mentioned only included patients with acute liver failure or ACLF (with a better prognosis for recovery or allocation of a donor-liver) but not patients with secondary liver failure (e.g. due to hypoxic hepatitis or to a refractory hyperbilirubinemia), excluded specific patient populations e.g. septic patients or patients with the need for renal replacement therapy. In these highly-selective study patient populations, there is still no clear benefit regarding morbidity and mortality compared to standard medical therapy. Our data shows, that clinical use of ELS in Germany as a therapy option for acute liver dysfunction (ALF, AoCLF or decompensated liver cirrhosis) is obviously heterogeneous regarding indications and performed in patients with a high a-priori mortality.

Thus, we agree with individual authors [2] as well as the current EASL (European Association for the study of the liver) [19] and AGA (American Gastroenterological Association) [20] recommendations, who suggest, that ELS should be restricted to clinical trials until patient populations with a defined benefit in morbidity and mortality are clearly identified.

### Strengths and weaknesses

Our epidemiological study using a nation-wide data set has some relevant limitations.

First, the incidences of ELS usage might not be transferable to other high-income countries due to limited access to extracorporeal therapy or limited reimbursement by the responsible health system providers. In Germany, ELS therapies are not restricted to specific tertiary care or transplant centers but may be performed by any ICU. Additionally, due to data protection restrictions, no further detailed analysis was allowed regarding care-provider-specific mortality. As with many other epidemiological studies, correlations of mortality rates between subpopulations are no causations.

We are unable to provide mortality rates for the subpopulations of patients with ALF, AoCLF or decompensated liver cirrhosis without ELS therapy. This is related to the fact, that these definitions are disease states of specific underlying entities which can be recorded within the DRG-System with a larger number of codes. This is comparable to the limitation of a recently published study by Karagiannidis et al. who explored the mortality of ECMO (extracorporeal membrane oxygenation) patients but were unable to provide mortality rates for ARDS due to different codings of the respective underlying disease [21]. Subgroup analysis of” primary acute liver dysfunction” was performed by grouping patients with the appropriate ICD. However, as the secondary liver failure related main and secondary diagnoses are extremely heterogeneous, use of OPS coding of major cardiac surgery was used to identify the patient subset of secondary liver failure (as patients with acute liver dysfunction will only rarely undergo cardiac surgery within the same hospital stay, an acute liver dysfunction was assumed to be a secondary liver failure after cardiac surgery). Nevertheless, this heuristic approach had to be applied, as the data source does not provide information regarding temporal sequence of OPS and ICD coding. Furthermore, the specific underlying hepatic pathology (e.g. hypoxic hepatitis, refractory hyperbilirubinemia) in the cardiothoracic subpopulation cannot be extracted from the data. Summing up the numbers of ELS therapies in both “primary” and “cardiac” grouping, almost 80–94% of annual ELS cases in Germany are covered. This underlines the appropriateness of our approach to define the specific subgroups.

Thus, as mentioned above, real incidences of acute liver dysfunction cannot be estimated from this data set. Additionally, our federal statistics office data set does not allow to explore the specific pathologies of liver dysfunction that resulted in a case-related ELS therapy in the primary nor secondary liver dysfunction subpopulations. Thus, the indications for ELS in the cardiothoracic subpopulation are speculative (e.g. hypoxic hepatitis, persistent hyperbilirubinemia). Further studies in the future should explore these issues.

Underreporting bias might be another relevant limitation. Extracorporeal organ support therapies as well as surgical procedures (e.g. transplantations, cardiothoracic surgical procedures) are expensive. Therefore, a relevant underreporting bias by the care provider might only be theoretical as correct documentation of these expensive procedures are in the interest of the institution to receive financial reimbursement. On the other hand, evaluation for liver transplantation can be documented using specific coding as shown above. However, as these case-specific evaluations do not result in relevant reimbursement, a relevant underreporting bias might have occurred. We were unable to separate data sets of patient cases with ELS use before (“bridge-to transplant”) and after liver transplantation (graft dysfunction) as the data source does not provide exact time points for performed procedures within a single case. As above, future observational trials should investigate this relevant liver transplantation topic.

The numbers of ELS sessions per case cannot be extrapolated from the data set as there are no specific DRG-codes for this question. Additionally, we are only able to provide data for case-related in-hospital mortality but not for other time spans (e.g. 30-day mortality, one-year mortality). Last, we are unable to provide data of hospital-specific ELS utilization as the data source provider did not allow us to evaluate this question due to data protection laws and the low annual number of ELS cases per hospital.

## Conclusions

In conclusion, there was a continuous utilization of extracorporeal liver support therapy in Germany between 2007 and 2015. Since 2012, more than 50% of all annual ELS cases were performed in the context of cardiac surgery. ELS was rarely used in a liver transplantation setting (bridge-to-transplant / graft dysfunction), as the majority of patients was not even evaluated for or received a liver transplantation. Mortality rates were higher in the German “real-world” patient population compared with international RCT study settings. Male patients as well as patients with secondary causes of acute liver dysfunction in the context of cardiothoracic surgery had a higher mortality rate compared to female patients or patients with primary liver failure (ALF, AoCLF, decompensated liver dysfunction). Future studies should focus on the identification of specific patient populations in which an extracorporeal liver support therapy might be beneficial. Contrary, patient populations with a high level of mortality should be identified to avoid futile extracorporeal organ support therapy. We suggest, that extracorporeal liver support therapies should only be performed in the context of clinical trials.

## Data Availability

Full data sets are available via the corresponding author: wiesmann@med.uni-marburg.de

## Abbreviations

ALF: Acute liver failure
AoCL: Acute on chronic liver failure
DRG: Diagnosis related grouping
ECLS: Extracorporeal cardiac life support
ECMO: Extracorporeal membrane oxygenation
ELS: Extracorporeal liver support
FPSA: Fractionated plasma separation; absorption and dialysis
ICD: International classification of diseases; intensive care unit
MARS®: Molecular Adsorbent Recirculating System
OPS: Operations and procedures
